# An optimal control theory approach to non-pharmaceutical interventions

**DOI:** 10.1186/1471-2334-10-32

**Published:** 2010-02-19

**Authors:** Feng Lin, Kumar Muthuraman, Mark Lawley

**Affiliations:** 1 School of Biomedical Engineering, Purdue University, 206 S Martin Jischke Drive, West Lafayette, IN 47907-2032, USA; 2 CBA 5.234, Dept of Information, Risk, and Operations Management, Austin, TX 78712, USA

## Abstract

**Background:**

Non-pharmaceutical interventions (NPI) are the first line of defense against pandemic influenza. These interventions dampen virus spread by reducing contact between infected and susceptible persons. Because they curtail essential societal activities, they must be applied judiciously. Optimal control theory is an approach for modeling and balancing competing objectives such as epidemic spread and NPI cost.

**Methods:**

We apply optimal control on an epidemiologic compartmental model to develop triggers for NPI implementation. The objective is to minimize expected person-days lost from influenza related deaths and NPI implementations for the model. We perform a multivariate sensitivity analysis based on Latin Hypercube Sampling to study the effects of input parameters on the optimal control policy. Additional studies investigated the effects of departures from the modeling assumptions, including exponential terminal time and linear NPI implementation cost.

**Results:**

An optimal policy is derived for the control model using a linear NPI implementation cost. Linear cost leads to a "bang-bang" policy in which NPIs are applied at maximum strength when certain state criteria are met. Multivariate sensitivity analyses are presented which indicate that NPI cost, death rate, and recovery rate are influential in determining the policy structure. Further death rate, basic reproductive number and recovery rate are the most influential in determining the expected cumulative death. When applying the NPI policy, the cumulative deaths under exponential and gamma terminal times are close, which implies that the outcome of applying the "bang-bang" policy is insensitive to the exponential assumption. Quadratic cost leads to a multi-level policy in which NPIs are applied at varying strength levels, again based on certain state criteria. Results indicate that linear cost leads to more costly implementation resulting in fewer deaths.

**Conclusions:**

The application of optimal control theory can provide valuable insight to developing effective control strategies for pandemic. Our findings highlight the importance of establishing a sensitive and timely surveillance system for pandemic preparedness.

## Background

Emerging influenza is threatening the world with the next pandemic [1]. The current swine flu caused by a novel H1N1 virus has infected a documented 182,166 humans, killing 1,799 from April 2009 to August 2009 [2]. The World Health Organization (WHO) declared the outbreak to be a pandemic because of growing worldwide cases [3]. Currently, the severity of the outbreak is moderate as most people recover from infection without the need for medical care [4]. However, if the virus mutates and achieves the ability to cause severe illness, it will kill more people and overwhelm the health system. Vaccination is the most effective means of pandemic mitigation. Vaccine production is a complex multi-step process which involves development, manufacturing, and delivery processes and current levels of vaccine production capacity are inadequate. Thus many uncertainties exist in every step and effective vaccines are typically available well after the viral strain has emerged [5-8]. For instance, vaccines against the H1N1 strain are still under development and will remain in short supply by November 2009 [9]. Current stockpiling of antiviral drugs will also be in short supply and their efficiency will be limited once a pandemic occurs [7,8]. Public health systems need to be prepared for cases when effective pharmaceutical interventions are unavailable. Non-pharmaceutical interventions (NPIs) are necessary to delay and dampen the pandemic before pharmaceuticals become available [7]. Recommended NPIs include: (1) social distancing: school closure, workplace distancing, restricted public gathering and travel; (2) case containment measures: voluntary case isolation, voluntary quarantine of members of households with ill persons; and (3) infection control measures: hand hygiene, cough etiquette, and mask/respirator usage [1,7].

NPIs were implemented during the 1918 pandemic and more recently during the severe acute respiratory syndrome (SARS) outbreak of 2003. Although research on these events confirms the importance of NPIs, sub-optimal triggering during the 1918 pandemic rendered NPIs only moderately effective at reducing mortality [10-12]. During SARS, sheltering and quarantine were found to be effective [13,14], while border screening was not [6]. During the current H1N1 outbreak, infection control is recommended to prevent spread of the virus among humans. Public health authorities are developing action plans which may request social distancing actions depending on the severity of the outbreak [15].

Mathematical models are often used to study disease spread, with the Susceptible-Infectious-Recovered (SIR) model being preferred for diseases spread via droplet and aerosol. The SIR model has been used to study pandemic flu [11,12,16-25], seasonal flu [26-28], SARS [13,29-31], and smallpox [32-35]. These papers use SIR to simulate the disease outbreak and evaluate the effectiveness of selected control measures under various predefined scenarios. They do not provide optimal controls for initiating implementation, and thus we will not review them here. SIR literature most directly relevant to this work includes [36-41]. These authors use the SIR model to study optimal controls, i.e., controls that minimize a prescribed objective function. Most show "bang-bang" controllers to be optimal (in the sense of minimizing a specified objective function). These policies apply no control until the occurrence of a triggering event and then apply controls at maximum strength.

Sethi derived optimal closed-form results for isolation and immunization policies [37,38] using an SI model. With this model, the population is partitioned into two parts, susceptible and infectious. The control is to either isolate and vaccinate at a maximum rate or do nothing. Infectious individuals who recover become susceptible once again, and thus immunity due to infection and subsequent recovery are not considered. Clancy [36] studied the properties of optimal policies for isolation and immunization assuming that all infectious individuals can be immediately isolated and all susceptible individuals can be immediately immunized. The policy takes no action when the number of infectious is below an optimal threshold and immediately isolates and/or immunizes when the number exceeds the threshold. However, they can only obtain optimal policies when the state space is small. Morton and Wickwire [40] developed optimal control policies for immunization assuming an infinite pandemic terminal time. However their switching curve derivation has an error in the derivatives (Eqs. 7a and 7b of [40]) and thus their results are unclear. Behncke [41] derived mathematical properties of optimal vaccination programs under the following assumptions: 1) the time when vaccine becomes available is known; 2) infectious individuals can be immediately and completely isolated; and 3) the time horizon of the pandemic is infinite.

Overall, we think the underlying assumptions of currently published results are questionable. It is not the case that all infectious can be immediately identified and isolated. Further, planners do not know when vaccines will become available, and even when available, it is not true that mass prophylaxis is instantaneous. Finally, during a pandemic, people will die. The current models do not account for mortality, which could be significant for viral strains such as H5N1.

In this work, we use an expanded SIR model to develop triggers for NPI implementation to minimize expected person-days lost resulting from influenza related deaths and NPI implementation. NPI policies are derived for a deterministic control model. Results are compared with the most relevant optimal control papers discussed above. Multivariate sensitivity analyses based on Latin Hypercube Sampling are performed to investigate the effects of input parameters on the control policy structure and the mean cumulative deaths. Additional studies investigate the effects of departures from the modeling assumptions, which include exponential terminal time and linear NPI implementation cost.

## Methods

### Optimal control model

**Figure 1:**
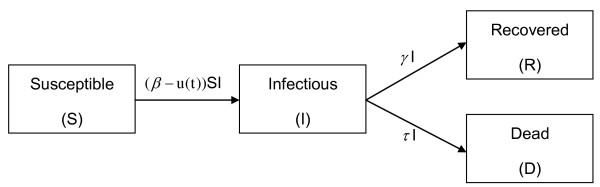
**Scheme of Susceptible-Infectious-Recovered/Death (SIRD) Model**. Boxes represent compartments and arcs represent flux between compartments. Figure 1 expands the classic Susceptible-Infectious-Recovered (SIR) model to capture the mortality.

In this section, we formulate an optimal control problem with an expanded epidemic model to compute NPI implementation strategy. To understand the following discussion, the reader is referred to Figure 1, which illustrates the compartmental model, and to Table 1, which provides a summary of notation. To construct the model, we make six assumptions:

**Table 1:** Model notation. provides a summary of notation.

List of notation	
*s*(*t*)	proportion of population that is susceptible in the community at time *t*, *s*(*t*) ∈ [0, 1]
*i*(*t*)	proportion of population that is infectious in the community at time *t*, *i*(*t*) ∈ [0, 1]
*r*(*t*)	proportion of population that has recovered in the community at time *t*, *r*(*t*) ∈ [0, 1]
*d*(*t*)	v of population that has died in the community at time *t*, *d*(*t*) ∈ [0, 1]
*x*(*t*) = (*s *(*t*), *i*(*t*), *r*(*t*), *d*(*t*))	state that describes the disease status of a community
*x*(0) = (*s *(0), *i*(0), *r*(0), *d*(0))	initial disease state of a community
*u*(*t*)	decision variable to model NPI implementation, *u*(*t*) ∈ [0, *b*]
*b*	maximum reduction in infection rate *β *by NPI implementation, *b *∈ [0, *β*]
*T*	time when vaccine becomes available, assumed to be exponential with mean Φ
*β*	infection rate
*γ*	recovery rate
*τ*	death rate
*c*	relative cost of NPI compared to a single death, *c *∈ [0, 1]
*R*_0_	basic reproductive number, the average number of secondary cases an infectious individual case will cause
*V *(*x*; *u*)	value function defined as expected person-days lost
	control that minimizes the value function
*ψ *(*s*, *i*)	switching curve
Ω ={(*s*, *i*); *s*, *i *≥ 0, *s *+ *i *≤ 1}	state space
Ω1 = {(*s*, *i*) ∈ Ω, u* > 0}	state space where u* > 0
Ω2 = {(*s*, *i*) ∈ Ω, u* = 0 }	state space where u* = 0
	proportion of the control space
*HJB*	Hamilton-Jacobi-Bellman equation

1. At any time, *t *≥ 0, the community is composed of *S*(*t*) susceptible, *I*(*t*) infectious, *R*(*t*) recovered, and *D*(*t*) deceased individuals. The population is closed, ignoring the demographic turnover or immigration, i.e. *S*(*t*) + *I*(*t*) + *R*(*t*) + *D*(*t*) = *N*. To make the analysis independent of population size, we normalize the model by letting , , , and . Thus, any community can be described by state variable *x*(*t*) = (*s*(*t*), *i*(*t*), *r*(*t*), *d*(*t*)).

2. The population is homogeneously mixed and people make contact at random.

3. People susceptible are able to get infected when they contact infectious people. Once infected, they move into the infectious compartment. People infected can either recover at a constant rate, *γ*, or die at a constant rate, *τ*. People recovered are assumed to be immune within our study horizon.

4. NPI implementation is modeled by the decision variable *u*(*t*), where 0 ≤ *u*(*t*) ≤ *b *<*β*. NPI implementation reduces the rate of contact between susceptible and infectious individuals, and thus dampens the infection rate from *β *to a lower level, *β *- *u*(*t*). In this paper, we only consider NPIs that will disrupt the normal societal functions and result in significant economic impact, such as school closure, work space distancing and voluntary isolation and quarantine. We assume these NPIs have the same effect in reducing disease spread.

5. We let person-days lost due to death be unit cost, while *c *≤ 1 will be person-days lost due to NPI implementation. The cost *c *is a weight of NPI cost relative to death. It measures the loss of productivity (person-days) due to implementing NPIs. To determine the value of *c*, the public health officials need to consider many factors, such as culture of the community, perceptions to death, consequences of pandemic and of the NPIs, economic constraints, and etc. We assume that cost of NPI implementation increases with implementation strength. In particular, we consider two cost structures, a linear structure in which cost is proportional to NPI strength, and a quadratic structure in which the change in cost with NPI strength is more variable. This paper is focused most extensively on the linear structure.

6. The optimization horizon *T *is assumed to be the time when effective vaccine becomes available to the community. Although the production of the first doses of vaccine is estimated to take 4-6 months once the virus strain can be identified, nobody knows exactly when circulating virus can be identified. Along with vaccine development, there are issues regarding manufacturing, delivery, and deployment. To capture the uncertainty in vaccine arrival time, we let *T *follow an exponential distribution. We can derive a model with a more general vaccine arrival time, but the problem is not currently numerically tractable, that is, algorithms for the general terminal time case present significant challenges and yet to be developed and tested. The exponential assumption ensures we get the discounted value function, which allows for a solution and some insights about the policy. In later sections we tested the model sensitivity to the exponential assumption. The probability density function (pdf) of *T *is written as *f*(*T*, Φ) = Φ*e*^-Φ*T*^, where *T *≥ 0.

Because NPI implementation disrupts daily activities vital to a productive society, they should only be used when they will be effective. Thus, in developing triggers, NPI cost must be balanced with NPI effectiveness. In this work, we use person-days lost to measure NPI cost and effectiveness. NPIs cause person-day losses by shutting down vital activities, but mitigate person-day losses by reducing mortality. By capturing the dynamic relationship between NPI implementation, mortality, and associated person-day losses, our model provides a mechanism for finding an NPI triggering scheme that helps minimize total person-days lost during the time period before vaccines become available.

The dynamics of the controlled epidemic are given by the non-linear differential equations (dots denote time derivatives, i.e. :(1)

where *s*(*t*) + *i*(*t*) + *r*(*t*) + *d*(*t*) = 1, *s*(*t*), *i*(*t*), *r*(*t*), *d*(*t*) ≥ 0, and the control, *u*(*t*), takes values in [0, *b*]. The total person-days lost for a pandemic under a policy which prescribes an admissible control, *u*, will be denoted by the value function *V *(*x*, *u*), which is defined as:(2)

The objective is to minimize expected person-days lost over the time horizon, *T*, which is assumed to be the time when effective vaccine becomes available to the community. An admissible control, u*, which minimizes *V *(*x*, *u*) will be called optimal, and the corresponding value function is denoted by:(3)

### Model analysis

From Eqs. (1) and (3) we can see that the knowledge of the state variables *s*(*t*) and *i*(*t*) determines the other two, thus we focus our analysis on the susceptible and infectious compartments. This reduces the system to two dimensions, S and I. Thus, we study the control system in the (*s*, *i*)-plane, defined as Ω = {(*s*, *i*), *s*, *i *≥ 0, *s *+ *i *≤ 1}. We let (*s*, *i*) represent the initial state *x*(0) = (*s*(0), *i*(0)), and *V *(*x*, *u*) = *V *((*s*, *i*); *u*) be the associated value function, which captures total person-days lost starting in state (*s*, *i*) ∈ Ω and operating under control *u*. The expected value of V*(*s*, *i*) is calculated by integrating *V *((*s*, *i*), *u*)·*f*(*T*; Φ) over the range of T:

By changing the order of integration, the problem is converted to an infinite horizon discounted problem:(4)

Based on Pontryagin's Maximum Principle [42], which gives necessary conditions for optimal control, we can derive a first-order partial differential equation satisfied by the optimal value function, V*. This is called the Hamilton-Jacobi-Bellman (HJB) equation:(5)

where  and  denote the gradient of V* at the point (*s*, *i*).

Eq. (5) is a linear function of *u*, thus the optimal control will be "bang-bang" control [43], i.e., the candidate control, u*, should satisfy:(6)

where the switching curve *ψ *(*s*, *i*) is the coefficient of *u *in Eq. (5), defined as:(7)

The optimal control u* is pushed to its lower/upper bound depending on the sign of its coefficient, the switching curve *ψ *(*s*, *i*), because the HJB equation, Eq. (5), is linear in the control, *u*. However, if the HJB is nonlinear in *u*, the optimal control will not be bang-bang. An example will be the linear quadratic optimal control problem, where the optimal policy is a linear state feedback [42].

By the bang-bang nature of candidate u*, the NPIs should be implemented at the maximum level when *ψ *(*x*) *<*0. To understand this, assume the community is in state *x*_*A *_= (*s*, *i*) and consider exerting an instantaneous and small control, *δ*_*u*_, which will move the system to state *x*_*B *_= (*s *+ *δ*_*u*_*si*, *i*-*δ*_*u*_*si*) instantaneously. The exertion will cost *csδ*_*u *_person-days lost. If V*(*x*_*A*_)-V* (*x*_*B*_) exceeds *csδ*_*u*_, then it is cost-effective to implement the NPIs. Here, V* (*x*_*A*_)-V* (*x*_*B*_) is the rate of change of the value function V* in the (-*δ*_*u*_*si*, *δ*_*u*_*si*) direction, i.e., ). Hence,  indicates that exerting control reduces person-days lost. We keep implementing NPIs until *ψ *(*x*) > 0.

Due to the complexity of the Eq. (5), the switching curve, *ψ *(*x*), has no closed form. To find *ψ *(*x*), we use an algorithm based on Dynamic Programming [44], which requires discretization over time and space. The uniqueness and convergence of the solution is guaranteed by the viscosity solution concept developed in [44].

## Results

### NPI policies assuming linear NPI implementation cost

**Figure 2:**
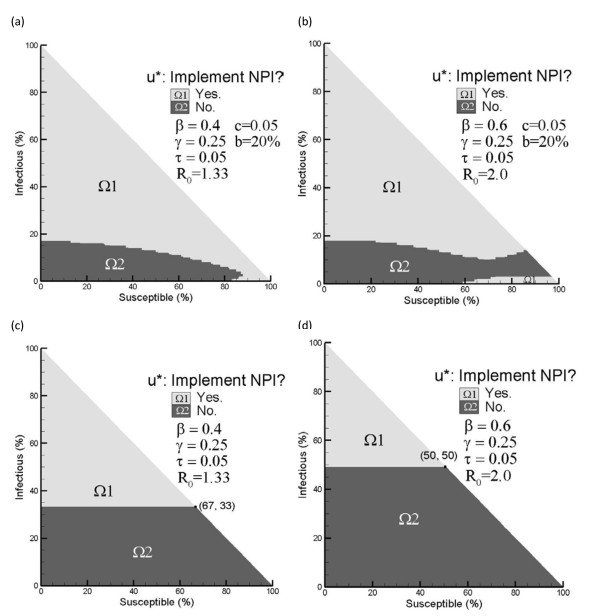
**Optimal NPI policy and optimal isolation policy derived in **[37]**(*γ *= 0.25, *τ *= 0.05, *c *= 0.05 and *b *= 0.2*β*)**. Figure 2 shows the optimal control policies for two infection rates, 0.4 and 0.6, given a recovery rate *γ *= 0.25 and a death rate *τ *= 0.05. 2(a) presents the optimal NPI control for *β *= 0.4 and *R*_0 _= 1.33. 2(b) presents the optimal NPI control for *β *= 0.6 and *R*_0 _= 2.00. 2(c) presents the optimal isolation policy derived in [37] for *β *= 0.4 and *R*_0 _= 1.33. (d) presents the optimal isolation policy derived in [37] for *β *= 0.6 and *R*_0 _= 2.00.

The optimal control, u*, is obtained by solving the HJB equation (Eq. (5)). Figure 2 shows u* for two infection rates, 0.4 and 0.6, given a recovery rate of 0.25 and a death rate of 0.05 (taken from [45]). Person-days lost from NPI implementation, *c*, is set to 0.05 (5% of the cost of a single death). The maximum impact of NPIs on the infection rate is assumed to be a 20% reduction. Note that the basic reproductive number *R*_0 _without any control is given by *R*_0 _= *β *= (*γ *+ *τ*).

Figures 2(a) and 2(b) indicate when to trigger NPI implementation. When the system state falls in region Ω1, NPIs should be implemented at maximum strength; in contrast, NPIs should not be implemented when the state falls in Ω2. For example, in the influenza scenario of Figure 2(a), NPIs should be implemented when 60% remains susceptible and 20% of the population is infected. However, if 50% remains susceptible and 10% is infected, it is better not to trigger the NPIs.

We compare our policy against the most relevant optimal isolation policies derived in [37]. Figures 2(c) and 2(d) show these isolation policies under the different infection rates. The control either isolates at a maximum rate when the number of infectious exceeds a threshold or does nothing. For a pandemic with *β *= 0.6 and *R*_0 _= 2.0, Figure 2(d) tells us not to act until the percent infectious exceeds 50%; while Figure 2(b) tells us to implement NPIs at an earlier stage of the outbreak, for example 99% susceptible and 1% infectious.

**Figure 3:**
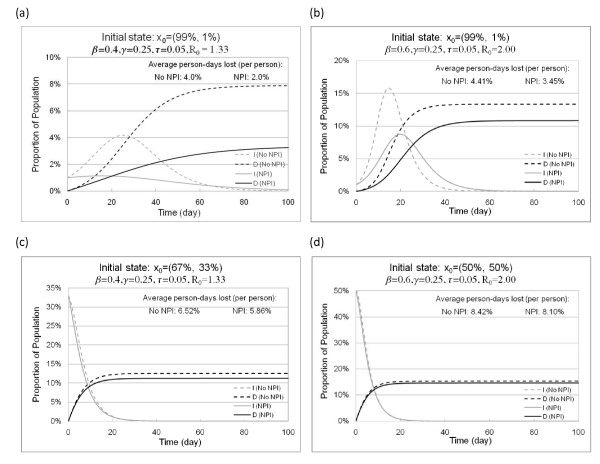
**Epidemic curves of infectious and dead population with and without NPI implementation**. Figure 3 shows the impact of optimal control on pandemic severity, peak, and total deaths, when NPIs are triggered at different initial states. (a) compares the epidemic curves with and without NPIs, starting from a state 99% susceptible and 1% infected when *β *= 0.4. (b) compares the epidemic curves with and without NPIs, starting from a state 99% susceptible and 1% infected when *β *= 0.6. (c) compares the epidemic curves with and without NPIs, starting from a state 67% susceptible and 33% infected when *β *= 0.4. (d) compares the epidemic curves with and without NPIs, starting from a state 50% susceptible and 50% infected when *β *= 0.6.

Figure 3 provides examples of the impact of NPIs triggered at different initial states. The figure presents graphics for three initial states. The initial state of Figures 3(a) and 3(b) is a triggering state for the NPI controls in Figures 2(a) and 2(b), while the initial states of Figures 3(c) and 3(d) are triggering states for the controls in Figures 2(c) and 2(d).

We illustrate cases for which *R*_0 _> 1, i.e., the uncontrolled infection spreads rather than dying out. Figures 3(a) and 3(b) compare the epidemic curves with and without NPIs, starting from a state 99% susceptible and 1% infected. According to Figures 2(a) and 2(b), the NPIs should be triggered at this state. In Figure 3(a), NPI implementation not only reduces the total death by 62%, but also eliminates the peak of the outbreak. Overall, NPI implementation saves 50% of the average person-days lost. In Figure 3(b), where a more severe pandemic is considered, the reduction in total deaths is 19% and NPI implementation reduces and delays the peak of outbreak, which allows additional time for vaccine development.

Figures 3(c) and 3(d) compare the epidemic curves with and without NPIs starting from states that fall on the control thresholds of [37] shown in Figures 2(a) and 2(d). The proportions of recovered and dead population were set to 0 because they could not be differentiated from infectious people in an SI model. It is still best to implement NPIs at these states but the impact is limited.

### NPI policies assuming quadratic NPI implementation cost

We also computed control policies for systems assuming quadratic control cost instead of linear cost, i.e., the value function is written as , while the system dynamics still follow Eq. (1). Based on Pontryagin's Maximum Principle [42], we can derive the Hamilton-Jacobi-Bellman (HJB) equation for the new problem:(8)

Eq. (8) is now a quadratic function of *u*, thus the optimal control will not be "bang-bang" control. The candidate control u* should satisfy:(9)

**Figure 4:**
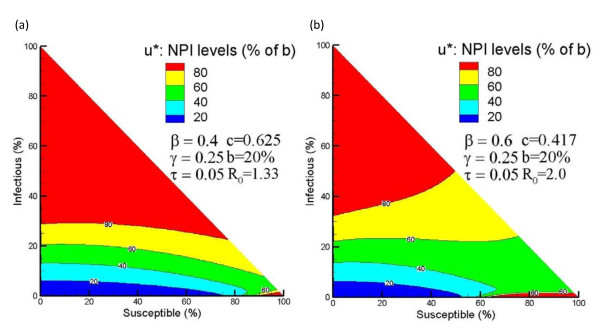
**Optimal NPI policy obtained under quadratic control cost**. Figure 4 presents the optimal NPI policy obtained under quadratic control cost. (a) presents the optimal NPI policy assuming quadratic control cost for an influenza pandemic characterized as *β *= 0.4, *γ *= 0.25, *τ *= 0.05, *c *= 0.05, and *b *= 0.2*β*. (b) presents the optimal NPI policy assuming quadratic control cost for an influenza pandemic characterized as *β *= 0.6, *τ *= 0.25, *γ *= 0.05, *c *= 0.05, and *b *= 0.2*β*.

By solving Eq. (8), we obtained the NPI policies for systems assuming quadratic control cost shown in Figure 4. Figures 4(a) and 2(a) show analogous plots of the control policy under quadratic and linear costs for influenza characterized by *β *= 0.4 and *R*_0 _= 1.33. Figure 4(a) shows a multi-level policy in which NPIs are applied at varying strength levels based on certain state criteria. Note that "red" indicates > 80% NPI strength, "yellow" indicates 60 80% NPI strength, and so forth. The level of NPI policy decreases from 100% of maximum NPI level to 0% as system state traverses from the upper to the lower part and from the right corner to the interior region. The contour line at u* ≈ 50% of maximum impelmentation level is very similar in shape and location to the boundary between Ω1 and Ω2 in Figure 2(a). As another demonstration, Figures 4(b) and 2(b) show the control policies under both control costs with influenza characterized by *β *= 0.6. The two boundaries between Ω1 and Ω2 in Figure 2(b) resemble the contour lines u* ≈ 50% of maximum impelmentation level in Figure 4(b). The two regions of high level of u* in the upper corner and lower right corner of Figure 4(b) correspond to two control regions Ω1 in Figure 2(b). In addition, Table 2 compares the means of the expected person-days lost per person due to death, , and control intensity, , between the linear and quadratic models. The overall NPI strength of the linear model is higher than that of the quadratic model, while the expected person-days lost due to death is lower. The linear model tends to implement NPIs more intensely and save more lives while having a higher overall cost.

**Table 2:** Comparison of the means of the expected person-days lost per person due to death and control intensity between the linear and quadratic models

			**(% of *b*)**	
		
*β*	Linear	Quadratic	Linear	Quadratic
0.4	3.676	5.447	38.9	36.7
0.6	4.210	5.448	37.2	33.9

### Sensitivity analysis

A multivariate uncertainty and sensitivity analysis was performed to study the effects of input parameters on the control policy for the linear cost model. This analysis investigated the effects of five inputs (*R*_0_, *γ*, *τ*, *c*, *b*) on a performance measure, *ω*, defined as the proportion of the control space to the total state space, i.e., . There is no well-defined performance measure to evaluate the NPI policy, especially when the policy is defined in a 2-dimensional state space. We chose *ω *because it captures the overall intensiveness of NPI implementations. In addition, we investigated the effect of these parameters on the outcome of applying the control policy, defined as the mean cumulative death, *d*_*T *_. We simulated the SIRD system under the optimal policy starting from all state (*s*_0_, *i*_0_, *r*_0_, *d*_0_), where *s*_0 _> 80%, *i*_0 _*<*20%, and *d*_0 _= 0. The simulation was terminated at a randomly selected exponential terminal time, and we recorded and analyzed the cumulative number of deaths. The mean cumulative death was calculated by taking the average of cumulative deaths over all tested initial states.

Table 3 summarizes the estimated probability distribution functions (PDFs) of five input parameters, assuming the input parameters are statistically independent. The PDFs of influenza transmission characteristics (*R*_0_, 1/*γ*, and 1/*τ*) are estimated based on the 1918 pandemic [25]. Note that the infection rate *β *can be written as *R*_0_(*γ *+ *τ*). The effect of NPIs, *b*, was found to reduce the infection rate, *β*, by up to 30-50% in 1918 and in many cases the effect of NPIs was very limited [11]; thus we assume the impact of NPI implementation, *b*, follows Uniform(0,50%). We are not able to find any empirical data on the cost of NPI, *c*. This value is a relative cost, which depends on decision makers' perceptions of saving lives versus maintenance of daily societal functions. In our analysis we let *c *take the value from 0 to 0.25 uniformly, which would imply that the cost of four sessions of maximal NPI implementation is equivalent to the cost of one death.

**Table 3:** Parameter ranges. summarizes the estimated probability distribution functions (PDFs) of five input parameters, *R*_0_, 1/*γ*, 1/*τ*, *c *and *b*.

Parameter	Unit	Parameter Distribution
*R*_0_	cases per infectious individual	Gamma(4.56,0.31) [25]
1/*γ*	days	Weibull(2.8, 3.7) [25]
1/*τ*	days	Gamma(3.5, 3.4) [25]
*c*		Uniform(0, 0.25)
*b*	%	Uniform(0, 50) [11]

We sampled ranges of the parameters 1000 times using Latin Hypercube Sampling (LHS) to generate 1000 scenarios [46-48]. Then we conducted multivariate uncertainty and sensitivity analysis to determine the uncertainty in the performance measure that was due to the uncertainty in estimating the input parameters. The descriptive statistics for *ω *and *d*_*T *_are given in Table 4, which lists the mean, variance, median, minimum, and maximum of *ω *and *d*_*T *_.

**Table 4:** Descriptive statistics from the uncertainty analysis.

	Proportion of control area *ω*	Mean cumulative deaths *d*_*T*_
Minimum	0	0.63%
Maximum	98.7%	76.39%
Mean	21.4%	14.25%
Median	0.6%	12.56%
Variance	11.2%	74.81%

Table 4 shows the descriptive statistics for *ω *and *d*_*T *_.

**Figure 5:**
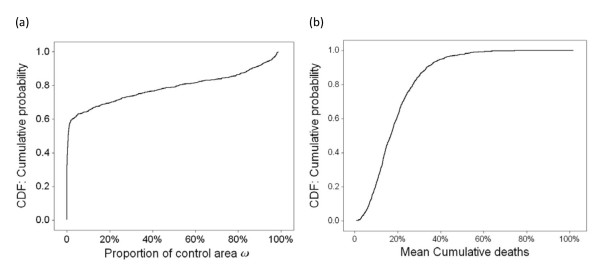
**Empirical CDFs for the proportion of control area and the mean cumulative death obtained from the 1000 LHS scenarios**. Figure 5 shows empirical cumulative distribution functions (CDFs) of *ω *and *d*_*T *_obtained from 1000 LHS scenarios. (a) shows the empirical CDF for the proportion of control area, *ω*. (b) shows the empirical CDF for the mean cumulative death, *d*_*T*_.

Figure 5 shows the empirical cumulative distribution functions (CDFs) of *ω *and *d*_*T *_obtained from 1000 LHS scenarios and Table 5 provides the partial rank correlation coefficients (PRCCs) for the performance measures and each parameter. The CDF of *ω *revealed a wide range of estimates due to the uncertainty in estimating the values of the five input parameters. Sixty percent of *ω *estimates are less than 2.3%, with a minimum of 0% and a maximum of 98.7%. For *ω*, the PRCCs are all statistically significant, i.e. *p *< 0.05. The cost of NPI implementation, *c*, the time when a death occurs after infection, 1/*τ*, and the time for an infectious person to recover, 1/*γ*, are the most statistically influential (|*PRCC*| > 0.5). An increase in *c *or 1/*τ *corresponds to a decrease in *ω*; while an increase in the infection period 1/*γ *corresponds to an increase in *ω*. The CDF of *d*_*T *_shows that the mean of cumulative death is 14.25%, with a minimum of 0.63% and a maximum of 76.39%. For *d*_*T*_, the PRCCs are are all statistically significant, i.e. *p *< 0.05. The most statistically influential inputs are 1/*τ*, *R*_0_, and 1/*γ *(|*PRCC*| > 0.5) while the other two parameters are lessinfluential. A decrease in 1/*τ *corresponds to an increase in *d*_*T*_; while an increase in *R*_0 _or 1/*γ *corresponds to an increase in *d*_*T *_.

**Table 5:** Partial rank correlation coefficients.

	Proportion of control area *ω*			Mean cumulative deaths *d*_*T*_		
		
Parameter	PRCC	p-value	Rank	PRCC	p-value	Rank
*R*_0_	0.182	< .0001	4	0.816	< .0001	2
1/*γ*	0.579	< .0001	3	0.747	< .0001	3
1/*τ*	-0.651	< .0001	2	-0.827	< .0001	1
*c*	-0.865	< .0001	1	0.342	< .0001	4
*b*	0.087	0.0059	5	-0.328	< .0001	5

Table 5 lists the partial rank correlation coefficients (PRCCs) for the performance measure *ω *and *d*_*T*_.

### Sensitivity to exponential terminal time assumption

To test the sensitivity of our control policy to the terminal time assumption, we simulated the disease propagation and studied the outcome of applying our NPI policy to settings with an exponential and a gamma terminal time. For each flu scenario specified in the sensitivity analysis, a corresponding NPI policy can be obtained. We randomly selected a value from *Exponential*(0.0056) and a value *Gamma*(3, 59.5) as the vaccine arrival time (or simulation terminal time). The sampling was repeated 20 times for each scenario. Then, we simulated the SIRD system starting from an initial state applying the corresponding NPI policy. The simulation was terminated at the sampled vaccine arrival times and the cumulative deaths were recorded. A total of 210 initial states were selected, where *s*_0 _≥ 80% and *i*_0 _≤ 20%, for a total of 210, 000 simulations. We studied the difference in cumulative deaths under these two vaccine arrival assumptions. Table 6 lists the descriptive statistics of percentage difference in cumulative deaths for the same initial states at two terminal times.

**Table 6:** Descriptive statistics of difference in cumulative deaths at exponential and gamma terminal time.

	Difference in cumulative deaths
Minimum	0
Maximum	32.55%
Mean	3.49%
Median	1.89%
Variance	0.19%

Table 6 lists the summary statistics of difference in cumulative deaths at exponential and gamma terminal time.

Overall, the difference in cumulative deaths under exponential and gamma terminal times is small (*mean *= 3.49%). The distribution of difference in cumulative deaths is left-skewed, with 91.8% of these differences being less than 10%. There are a few cases where the cumulative deaths differ significantly (≥ 30%). These cases all started from initial states where only a small proportion people are infectious, i.e., *i*0 ≤ 1%, and the difference between the selected gamma and exponential terminal time exceeds 60% of the maximum of these two. For example, in a case where *i*_0 _= 1%, *S*_0 _= 95%, gamma terminal time = 235 days and exponential terminal time = 55 days, the difference in cumulative death is 32.04%.

## Discussion

### Effect of NPI policies on the epidemic

NPIs reduce and delay the spread of pandemic by moderating social contact between susceptible and infectious people. Because NPIs disrupt daily societal functions, it is important that they be implemented judiciously. This requires identifying effective initiating triggers, which is a challenging research task. Implementing NPIs will impede influenza spread; on the other hand, normal societal functions will be interrupted. The optimal control method takes both aspects into account and tries to find the best balance between them, given the decision makers relative weighting of the two.

Based on Figures 2 and 3, early implementation for moderate and severe pandemic is very important for NPIs to have impact on the outbreak and the impact is effective only if NPIs are implemented early. Late NPI implementation might still be optimal, but the impact is much less. For a severe pandemic, it is optimal to trigger NPIs at the beginning stage when susceptible population is large and infectious population is small. If we miss the beginning stage, it is not optimal to implement NPIs until the outbreak is significantly progressed. This is because once the pathogen achieves a certain level of infection, NPIs are not effective against it, and thus are not worth the cost. That is, the benefit of NPIs at a stage when the disease has progressed significantly is less than the cost of NPI implementation. This finding supports the CDC pandemic mitigation guidelines, which state that when the pandemic is Category 4 or 5, all NPIs are recommended for early implementation [7]. Furthermore, earlier NPI implementation reduces and delays the peak of the outbreak as illustrated Figures 3(a) and 3(b), which allows additional time for vaccine development. If a severe pandemic occurs, hospitals will experience an overwhelming influx of patients and need to operate at their surge capacities. Earlier NPI implementation can reduce the magnitude of infectious at the peak, which relieves some of the burden on hospitals and other health care infrastructures. In contrast, NPIs are not nearly as effective if disease has already spread into the community as the cases shown in Figures 3(c) and 3(d). In both cases, NPIs are triggered after the peak of the outbreak, where hospitals might have already been operating at their surge capacities for a few weeks. Both cases start at states falling on the control thresholds recommended in Figures 2(c) and 2(d)[37]. This finding indicates that the additional complexity of our model is warranted when compared with the SI model used in [37]. Timely and sensitive surveillance systems are key to successful application of the optimal control method as knowledge of both the pathogen characteristics and the community state are assumed. The surveillance systems should be able to identify the virus quickly and provide accurate estimates for parameters which characterize the severity of an influenza. The effectiveness of the control policy depends on the accuracy of these estimates, which include infection rate *β*, death rate *τ *and recovery rate *γ*. Once the control policy is computed, we also need to track the community state to determine if NPIs should be triggered. As early NPI implementation is found to be much more effective, we do not want to miss the beginning stage of the outbreak. Thus, the surveillance system should also estimate the community state, including the size of the infectious and susceptible populations.

### Sensitivity analysis

Our sensitivity analysis identified three important input parameters for determining the overall NPI intensity. The important parameters are the NPI cost, death rate, and recovery rate. For high NPI costs, it is not worthwhile implementing NPIs because the benefit is less than the cost. For higher death rates, the policy sacrifices daily societal functions to save lives. In contrast, when recovery rate is small, infected people recover more slowly and continue infecting susceptible people. Thus, more NPI implementation is required. These results suggest that an influenza virus with a high death rate and a small recovery rate requires early intensive NPI implementation, particularly when the community places a high value on avoiding death.

Cumulative death was most affected by the death rate, the basic recovery number and the recovery rate. For higher death rates, a higher proportion of infected people will die. For higher basic reproductive number, more people will be infected, resulting in more deaths even when the death rate is smaller. For lower recovery rate, infected people recover at a slower rate, and thus more people will be infected. This suggests that an influenza virus with a high death rate, a high basic reproductive number and a small recovery rate is less affected by NPI implementation. NPI cost does not seem to affect the cumulative death. However, NPI cost was identified as the most influential (*PRCC *= -0.865) in determining the intensity of NPI implementation. Different communities have different perspectives of death versus disruption of daily societal functions. The range of *c *should be determined by decision makers after carefully evaluating the demographic, cultural, and economic characteristics of the community.

As a performance measure, *ω *does not capture the complete structure of the control policy. It only captures the overall control intensity, but subtle differences between control policies, such as distribution and shape of Ω_1 _in the state space, is missing. Policies that have different structure in the state space might have same *ω *value. Therefore, continued effort should be made in selecting more refined measures for the control policy performance.

### Linear v.s. quadratic cost function

If the cost function is linear, the control policy is bang-bang, which suggests implementing NPIs at the maximum strength or not implementing at all as shown in Figure 2. If the cost function is nonlinear, for example the quadratic cases presented in Figure 4, the control policy has multiple levels, which requires varying the NPI strengths as the system evolves from one state to another. It is easily shown from Eqs. 7 and 9 that if the linear model indicates NPI implementation for a state *x*, i.e., u* = *b *for state *x*, then the quadratic model also indicates some implementation in state *x*, i.e., u* > 0 for state *x*. But the inverse is not true. This property can be proved easily. If u* = *b *in the linear model, we have *ψ*(*s*, *i*) < 0, for all (*s*, *i*) ∈ Ω1 according to Eq. 7. So  < 0 holds for all (*s*, *i*) ∈ Ω1. Together with Eq. 9, we have u* > 0 for these states in the quadratic model.

Thus, a quadratic cost structure will require NPIs to be implemented in more states but with much lower intensity in those states. Overall, a linear cost structure leads to higher average NPI intensity than the quadratic cost. In the examples observed, the boundaries between control and non-control regions in linear model resemble the quadratic contour lines u* ≈ 50% of maximum implementation.

Moreover, the linear cost model appears to place more weight on death, thus implementing more control and saving more lives as shown in Table 2. In cases with quadratic cost structure, using a linear model might result in over-control which would not be marginally effective. However, quadratic cost introduces additional complexity in parameter estimation, computation, and policy interpretation. For example, we must determine how to implement NPIs at *X*% of maximum. This requires determining which NPIs will be implemented for each control level. The bang-bang policy, on the other hand, has only two levels, which is easier to understand and implement. Finally, it is not clear that a direct comparison between policies obtained under linear and qudratic cost structures is appropriate, because the value functions are defined differently. More research needs to be done to better define the NPI levels and interpret the policy if a non-linear cost function is chosen.

### Exponential vaccine arrival time

We are currently not able to derive an optimal control policy for a general terminal time assumption. Although the exponential optimal policy will not be optimal for cases with general terminal time, results shown in Table 6 indicate that the expected cumulative deaths predicted by the exponential optimal policy will be close to those occurring in a more general terminal time case. This suggests that the impact of the optimal exponential policy on virus spread is not always sensitive to terminal time distribution. Although the exponential assumption might not be completely realistic, making this assumption allows us to obtain the NPI policy, which then seems to provide desirable impact for cases with non-exponential terminal times.

### Model limitations

Our model was limited in several ways. First, although HJB can be derived for models assuming general terminal time (e.g., gamma), so far the control policy can only be computed assuming exponential terminal time. Second, the present modeling framework does not capture uncertainty in parameter estimation, i.e. the model accuracy relies on accurate estimation of input parameters. In practice, collection of accurate data and estimation of input parameters from data can be challenging and time consuming. Third, the present modeling framework assumes equal effect of various NPIs in a homogeneously-mixed population, while different NPIs will have distinct impacts for disparate population groups. Finally, bang-bang control must be further refined since it is not clear that on/off implementation is realistic for larger communities. To better apply optimal control methods in disease control problems, continued efforts should be made to refine the present model and to better estimate the input parameters.

## Conclusions

To conclude, we have considered a problem of non-pharmaceutical intervention (NPI) implementation for pandemic control using optimal control theory to develop triggers that minimize expected person-days lost associated with infection related death and NPI implementation over an exponential time horizon. The best control strategy for the model depends on the transmission characteristics of the influenza virus, the state of the pandemic, and the cost and implementation levels of NPIs.

We present the computed policies under different transmission characteristics, where it is optimal to activate all NPIs when the system state falls in the control region, Ω1. The optimal policy can be calculated for any combination of flu and cost parameters. We compare the impacts of NPIs triggered at different states, which supports the idea of early containment. For comparison, we present the NPI policies assuming quadratic control cost. The quadratic cost assumption introduces additional complexity into parameter estimation, computation and policy interpretation, thus more research needs to be done to better define the NPI levels and interpret the policy. We perform multivariate sensitivity analysis, which identifies important parameters that affect the intensity of control and the outcome of applying the policy. The findings highlight the importance of establishing a sensitive and timely surveillance system. Finally, we study the outcome of applying our NPI policy under exponential and gamma terminal times, and find small difference in the cumulative death.

Many uncertainties exist in estimating flu parameters, future research directions include developing a model that allows using stochastic rather than deterministic inputs and updates the control polices in real time. Since NPI implementation is not mandatory, compliance to NPI requirements is crucial for successful implementation. Community engagement, job security, and disruption of daily life affect compliance to NPI implementation [7]. Moreover, prolonged outbreak might result in compliance fatigue. Thus, in future work, we will integrate time-based compliance models into the system dynamics. Other important research directions include consideration of population heterogeneity, stochasticity and partial observability in disease outbreak, and developing methods for general terminal time distributions.

## Competing interests

The authors declare that they have no competing interests.

## Authors' contributions

LF conducted the research, including model design, acquisition of data, analysis and interpretation of data, and manuscript drafting. KM provided important guidance in model design and methods. He also revised the manuscript critically for important intellectual content. ML supervised the study. He had actively involved in model design and interpretation of data. He also revised the manuscript critically for important intellectual content. All the authors have given approval of the version to be published.

## Pre-publication history

The pre-publication history for this paper can be accessed here:

http://www.biomedcentral.com/1471-2334/10/32/prepub
